# Time-Resolved Label-Free Proteomics of SHK-1 Cells After *Renibacterium salmoninarum* Inoculation Reveals Early Host-Cell Remodeling

**DOI:** 10.3390/ijms27135773

**Published:** 2026-06-26

**Authors:** Jorge F. Beltrán, Jörn Bethke, Sandra Flores-Martin, Claudia A. Barrientos, Marcelo Aguilar, Adolfo Isla, Felipe Almendras, Marcos Mancilla, Alejandro J. Yañez

**Affiliations:** 1 Department of Chemical Engineering, Faculty of Engineering and Science, Universidad de La Frontera, Ave. Francisco Salazar 01145, Temuco 4811230, Chile; beltran.lissabet.jf@gmail.com; 2 BioChemIntelli, Bioinformatics Research & Development, Temuco 4780000, Chile; 3 Aquit SpA, Av. Libertad 269 of 904, Viña del Mar 2520000, Chile; jbethke@aquit.net; 4 Keybio Solution E.I.R.L., Valdivia 5090000, Chile; sandra_uach_2008@hotmail.com (S.F.-M.); claud.barrientosman@gmail.com (C.A.B.); marcelo.aguilarcart@gmail.com (M.A.); 5 Departamento de Ciencias Básicas, Facultad de Ciencias, Universidad Santo Tomas, Valdivia 5090000, Chile; adolfoisla@gmail.com; 6 Departamento de Investigación y Desarrollo, Greenvolution SpA, Puerto Varas 5550000, Chile; felipe.almendras@greenvolution.cl; 7 ADL Diagnostic Chile, Sector la Vara, Puerto Montt 5480000, Chile

**Keywords:** *Renibacterium salmoninarum*, bacterial kidney disease, SHK-1, *Salmo salar*, label-free quantitative proteomics, time-course analysis, temporal modules, regulated cell death

## Abstract

*Renibacterium salmoninarum*, the etiological agent of bacterial kidney disease, is a facultative intracellular pathogen whose interaction with salmonid phagocytic cells remains poorly resolved at the protein level. Here, we aimed to define the temporal protein-abundance architecture of SHK-1 macrophage-like cells after *R. salmoninarum* inoculation and to test whether this response supports broad canonical cell-death pathway engagement. We used label-free quantitative LC-MS/MS proteomics to profile SHK-1 cells over a 48 h post-inoculation time course. Because the design included a single non-infected T0 baseline, analyses were framed as baseline-referenced post-inoculation comparisons rather than a fully controlled mock time course. Of 6842 proteins retained for statistical modeling, 2254 were strictly differentially abundant in at least one contrast relative to T0 (adjusted *p* < 0.05 and |log_2_FC| ≥ 0.585). Perturbation was strongest at 1–2 h and progressively contracted at later time points. Among 1278 recurrent proteins, k-means clustering resolved four temporal modules capturing coordinated remodeling of lysosomal, immunometabolic, cytoskeletal, stress-response, and antioxidant programs. A curated cell-death panel spanning apoptosis, pyroptosis, necroptosis, ferroptosis, and PANoptosis yielded only three detected markers; CASP3 and MLKL met the strict threshold, whereas ACSL4 remained sub-threshold. Overall, early host-cell remodeling, rather than broad canonical death-program execution, was the predominant proteomic signature of SHK-1 cells during the first 48 h after *R. salmoninarum* inoculation.

## 1. Introduction

Atlantic salmon (*Salmo salar*) aquaculture is a major component of global fish production and of the economies of major producing countries such as Norway, Chile, and Canada [1]. Its sustainability, however, remains strongly constrained by infectious disease outbreaks that reduce animal performance, compromise welfare, and generate substantial economic losses [2]. Among the most persistent bacterial threats is *Renibacterium salmoninarum*, the etiological agent of bacterial kidney disease (BKD), a chronic granulomatous infection that primarily affects the kidney but can disseminate systemically to multiple internal organs [3,4]. BKD remains especially difficult to control because it combines vertical transmission, prolonged infection, immune evasion, and limited efficacy of currently available prophylactic strategies [3,4,5]. A central feature of this pathogen is its facultative intracellular lifestyle: survival within host phagocytes can promote dissemination while reducing exposure to humoral defenses [6]. For that reason, the biology of the infected host cell is not a peripheral aspect of BKD pathogenesis, but one of its core unresolved mechanistic layers.

Most previous molecular studies of salmonid responses to intracellular bacterial infection have been conducted at the transcriptomic level or through targeted gene-centered analyses [7,8]. These approaches have been informative for innate immune signaling, but they leave unresolved which changes are ultimately expressed at the level of protein abundance, where vesicular trafficking, lysosomal remodeling, metabolism, oxidative balance, and structural reorganization are executed. This distinction is important because mRNA abundance does not necessarily predict protein abundance owing to post-transcriptional control, protein turnover, and post-translational regulation [9,10,11]. It is also important because infection is inherently dynamic: the earliest hours after inoculation are likely to include bacterial uptake, host-cell stress, and compartmental reorganization, whereas later phases may reflect adaptation, recalibration, or persistent dysfunction. Proteomic and integrative omic approaches are therefore useful for connecting infection-associated cellular remodeling with protein-level changes in host–pathogen systems [12]. A time-resolved proteomic view is therefore needed to define not only whether the host proteome changes, but when the dominant layers of remodeling emerge and whether they remain stable or reverse over time.

Regulated cell-death pathways represent an important but technically challenging layer of this problem. Apoptosis, necroptosis, and pyroptosis are central components of the antimicrobial response to intracellular infection and can shape both pathogen restriction and inflammatory signaling [13,14]. More recent frameworks have expanded this landscape to include ferroptosis and integrative PANoptosis, emphasizing that host-cell fate during infection is often determined by intersecting rather than isolated death programs [15,16]. Intracellular pathogens, in turn, frequently manipulate these pathways to preserve an intracellular niche, delay elimination of the infected cell, or reshape the inflammatory environment [13]. In the specific case of *R. salmoninarum*, interference with host defensive programs has long been suspected [17], and recent work in Atlantic salmon has linked intracellular bacterial sensing to DDX41- and STING-associated pathways connected to regulated cell-death signaling [18,19]. Even so, the extent to which canonical cell-death-associated proteins are detectably remodeled during the early post-inoculation period remains unresolved, particularly when assessed by global total-protein proteomics rather than by targeted activation-state assays.

The SHK-1 cell line provides a relevant experimental system in which to address this question. Derived from Atlantic salmon anterior-kidney macrophage-like cells, SHK-1 has been widely used in salmonid host–pathogen research and offers a tractable model for studying pathogen internalization and early innate cellular responses under controlled in vitro conditions [20,21,22]. Yet, despite the relevance of both SHK-1 and *R. salmoninarum* for intracellular infection biology in salmonids, the temporal proteomic architecture of SHK-1 cells after bacterial inoculation remains poorly defined. This gap is particularly important for distinguishing broad early proteome perturbation from more selective late remodeling and for evaluating whether a cell-death-oriented interpretation is truly supported at the abundance level.

In the present study, we used label-free quantitative LC-MS/MS proteomics to characterize the post-inoculation trajectory of SHK-1 cells exposed to *R. salmoninarum* over a 48 h time course. Because the experimental design includes a single non-infected baseline condition at T0, the analysis is explicitly framed as a time-resolved post-inoculation comparison relative to baseline rather than as a fully controlled mock time course. We therefore addressed three linked questions: first, whether global proteome perturbation was extensive and temporally concentrated in the early post-inoculation phase; second, whether recurrent temporal modules could capture the dominant response architecture more effectively than isolated pairwise contrasts; and third, whether a curated panel of canonical cell-death pathways provided robust protein-level support or only sparse pathway-associated modulation within the global dataset. By combining strict differential abundance analysis, temporal module reconstruction, and a deliberately conservative targeted cell-death layer, we sought to provide a compact but mechanistically informative account of host proteome remodeling without exceeding the inferential limits imposed by the experimental design.

## 2. Results

### 2.1. Early Proteome Perturbation Defines the Dominant Baseline-Referenced Response

Across the 24 analyzed samples, 6842 proteins met the abundance and detectability criteria required for statistical modeling. Under the strict differential abundance definition (adjusted *p* < 0.05 and |log_2_FC| ≥ 0.585), 2254 unique proteins were significantly altered in at least one post-inoculation contrast relative to T0. Because the experimental design included only a single non-infected baseline condition, subsequent comparisons are interpreted as baseline-referenced post-inoculation changes. The strongest perturbation was concentrated at 1 h and 2 h post-inoculation, when 1281 and 1296 proteins, respectively, satisfied the strict threshold, with down-regulated proteins predominating at both time points.

**Figure 1:**
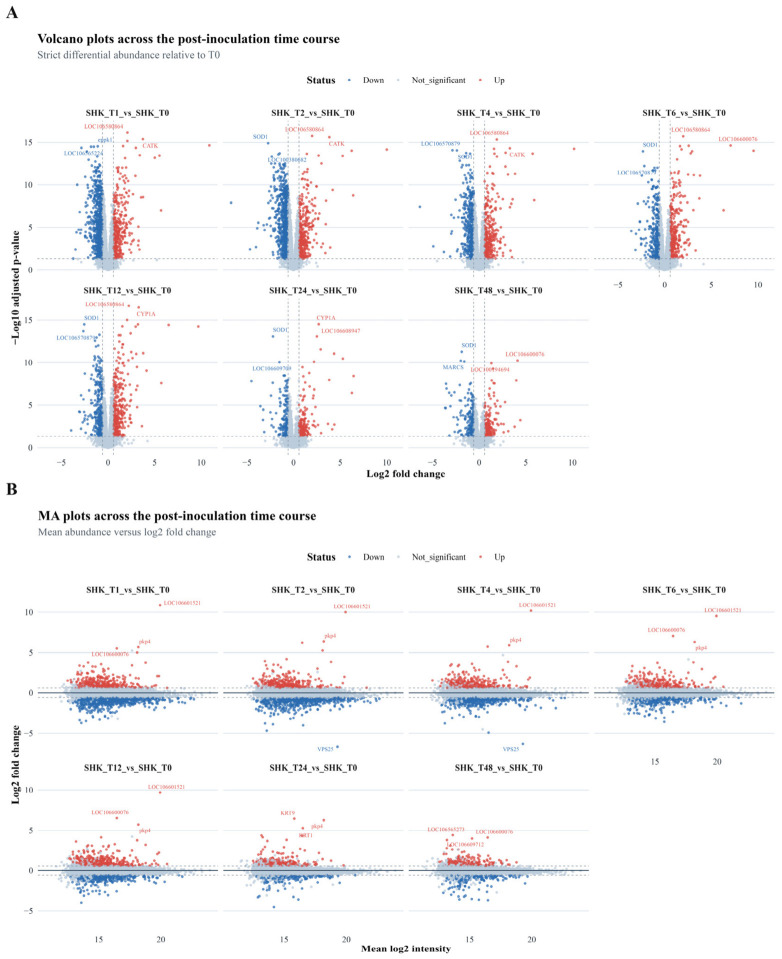
Global differential abundance landscape across the post-inoculation course. (**A**) Volcano plots for the seven post-inoculation contrasts relative to T0. Each point represents one quantified protein tested in the corresponding contrast, with colors indicating up-regulated, down-regulated, or non-significant proteins under the strict differential abundance definition (adjusted *p* < 0.05 and |log_2_FC| ≥ 0.585). Dashed vertical lines denote the fold-change cutoff and the horizontal dashed line denotes the adjusted significance threshold. Selected labels identify top-ranked significant proteins in each direction. (**B**) MA plots for the same contrasts, showing the relationship between average log_2_ intensity and log_2_ fold change. Horizontal dashed lines denote the strict fold-change cutoff, and labeled points identify proteins with the largest absolute abundance shifts in each contrast.

The number of altered proteins then declined to 902 at 4 h and 704 at 6 h, showed a moderate secondary increase at 12 h (775 proteins), and decreased further at 24 h and 48 h, where 432 and 391 proteins remained significant, respectively (Table 1). Volcano plots and MA plots likewise showed that the broadest effect-size dispersion and strongest statistical support were concentrated in the early contrasts, whereas later time points exhibited a narrower dynamic range and reduced amplitude of change (Figure 1A,B). As a complementary sample-level quality-control view, PCA of the normalized complete matrix (5034 proteins quantified across all 24 samples) showed a structured distribution across the first two principal components, with PC1 and PC2 explaining 32.2% and 16.7% of the variance, respectively (Supplementary Figure S1). PC1 separated the strongly perturbed early post-inoculation samples from T0 and the later 24–48 h samples, whereas intermediate time points occupied transitional positions; biological replicates generally grouped by time point, although some dispersion along PC2 was retained, and no sample was excluded as an outlier. Together, these results indicate that the main biological signal of the dataset lies in the early post-inoculation window and motivated a second analytical layer focused on recurrent temporal programs.

### 2.2. Recurrent Temporal Modules Resolve Coordinated Remodeling Across the Post-Inoculation Course

**Figure 2:**
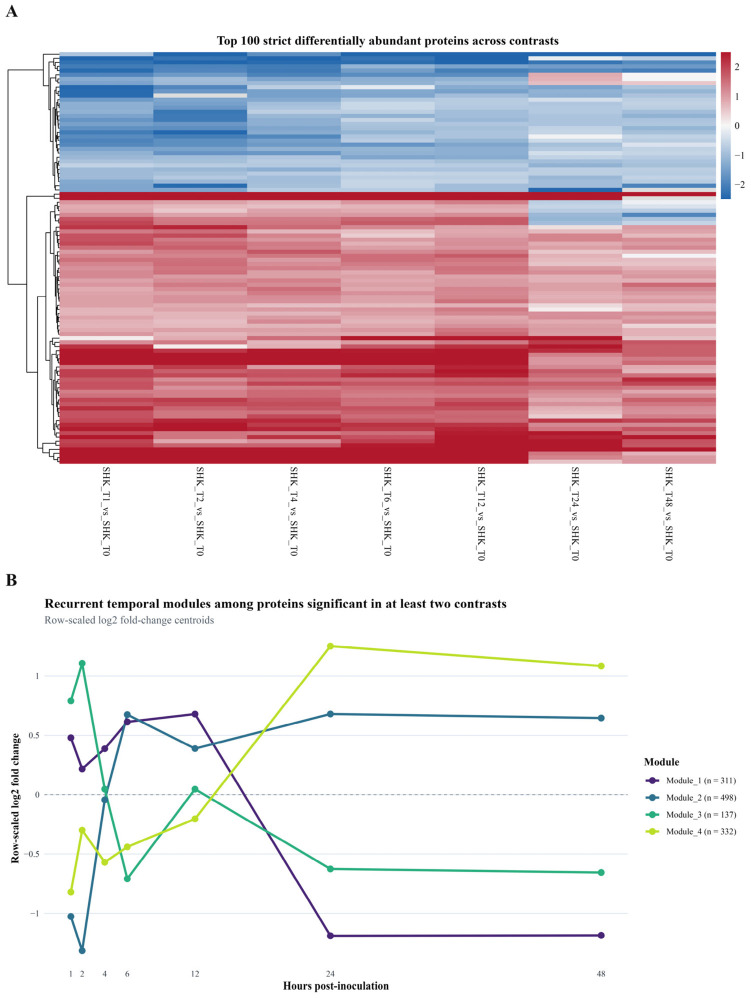
Temporal organization of strict differential abundance. (**A**) Heatmap of the top 100 proteins ranked by recurrence and effect size across the seven strict contrasts relative to T0. Cell values represent contrast-specific log_2_ fold changes, columns are ordered by post-inoculation time, and rows were hierarchically clustered to highlight dominant patterns of coordinated remodeling. (**B**) K-means-derived temporal modules summarizing the main row-scaled log_2_ fold-change centroid trajectories among the 1278 proteins that were significant in at least two contrasts. Module centroids were derived using four centers, 100 random starts, and a maximum of 200 iterations; module sizes are indicated in the legend.

To capture the dominant response architecture beyond isolated pairwise contrasts, we focused the module analysis on proteins that were significantly altered in at least two post-inoculation comparisons and had complete log_2_ fold-change trajectories across all seven baseline-referenced contrasts. This recurrence-filtered trajectory subset contained 1278 proteins and was used to reconstruct temporal profiles. Proteins significant at a single time point were not considered biologically irrelevant; rather, they were retained in the complete differential-abundance and recurrence summaries but excluded from k-means clustering because the goal of that layer was to model repeated temporal behavior rather than isolated events. The recurrence filter was direction-agnostic, so proteins with reversible patterns, such as early induction followed by late repression, could be assigned to temporal modules instead of being forced into monotonic categories. A heatmap of the top 100 strict differentially abundant proteins revealed a clear transition from broad early perturbation to more selective late-phase remodeling, with multiple proteins showing attenuation, reversal, or narrowing of response amplitude over time (Figure 2A).

K-means clustering resolved these trajectories into four recurrent temporal modules. Module 1 (*n* = 311) displayed positive centroids from 1 h to 12 h followed by a marked negative shift at 24–48 h. Module 2 (*n* = 498) was strongly repressed at 1–2 h and then progressively shifted toward positive values from 6 h onward. Module 3 (*n* = 137) captured transient early induction followed by a late negative phase. Module 4 (*n* = 332) remained negative through 12 h and then shifted sharply upward at 24–48 h (Figure 2B). To test whether the module labels were supported beyond representative proteins, we performed formal over-representation analysis using the 6842 modeled proteins as the background set.

This analysis provided formal support for the lysosomal interpretation of Module 1, which was significantly enriched for KEGG lysosome biogenesis (KEGG:04142, *p* = 2.43 × 10^−12^) and GO isoprenoid metabolic process (GO:0006720, *p* = 0.036). No GO, KEGG, or Reactome terms reached significance for Modules 2–4 after multiple-testing correction using the modeled protein background; therefore, these modules are interpreted descriptively based on their temporal profiles and recurrent high-effect representative proteins rather than as formally enriched functional categories. Representative proteins for Table 2 were selected to combine high recurrence across contrasts, large absolute log_2_ fold change, strong association with the assigned centroid, and interpretable functional annotation within each module. These proteins pointed to cytoskeletal and redox–stress remodeling in Module 2, stress and carbon metabolism in Module 3, and antioxidant/scaffold-associated programs in Module 4 (Table 2). Overall, the SHK-1 response to *R. salmoninarum* is better described as coordinated temporal remodeling than as a simple linear decline from an early peak. Supplementary Figure S2 and Datasets S3, S4 and S6 provide the complete heatmap, recurrence summary, module assignments, and functional enrichment results.

### 2.3. Canonical Cell-Death Pathways Show Only Limited Support at the Protein-Abundance Level

To assess whether the global remodeling identified above was accompanied by a strong signature of canonical cell-death execution, we applied a curated panel spanning apoptosis, pyroptosis, necroptosis, ferroptosis, and PANoptosis. This panel comprised 37 unique genes distributed across five pathway categories, and the full curated panel is provided in Supplementary Dataset S5. Only three curated gene symbols were detected in the proteomic dataset: CASP3, MLKL, and ACSL4.

Of these, CASP3 and MLKL met the strict differential abundance threshold, whereas ACSL4 was detected but remained below the predefined fold-change cutoff. CASP3 mapped to apoptosis, MLKL contributed to both necroptosis and PANoptosis, and ACSL4 represented the ferroptosis-associated component of the detected panel. At the pathway-summary level, detection coverage was sparse: apoptosis showed 1 detected gene out of 11 panel genes, necroptosis 1 out of 7, PANoptosis 1 out of 8, ferroptosis 1 out of 10, and pyroptosis 0 out of 10.

**Figure 3:**
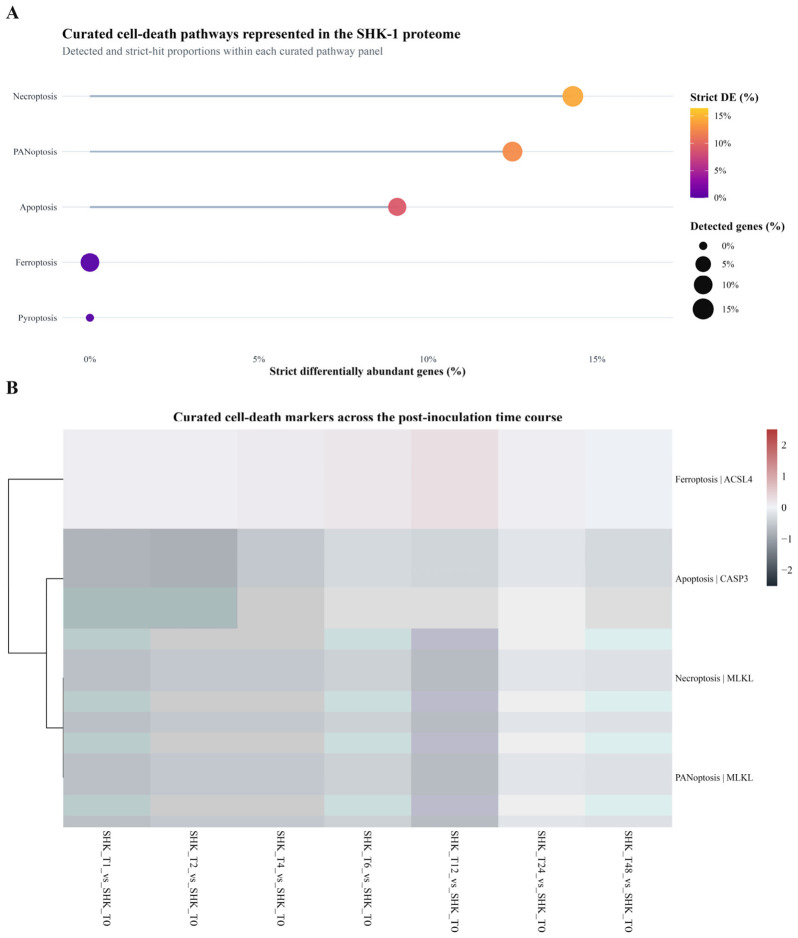
Targeted cell-death signatures across the post-inoculation course. (**A**) Pathway-level summary of the curated cell-death panel, which comprised 37 unique genes assigned across apoptosis, pyroptosis, necroptosis, ferroptosis, and PANoptosis. Point size indicates the percentage of panel genes detected within each pathway panel, whereas point color and horizontal position indicate the percentage meeting the strict differential abundance threshold. (**B**) Heatmap of detected cell-death markers across all seven post-inoculation contrasts relative to T0. Cell values represent log_2_ fold changes; when multiple matched proteins were assigned to the same pathway–gene combination, values were averaged across matched proteins within each contrast. Columns are ordered by time, and rows were clustered to summarize the limited pathway-associated signal captured in the global total-protein dataset.

Time-resolved profiles further showed that CASP3 and MLKL were predominantly characterized by negative log_2_ fold changes across the post-inoculation course, whereas ACSL4 remained close to baseline with only mild positive deviations (Figure 3A,B). CASP3 showed its largest strict decrease at 1 h (peak log_2_FC = −1.034) and remained a strict hit at 2, 4, and 48 h, while MLKL reached its strongest decrease at 12 h (log_2_FC = −0.685) and was also significant at 1 h. Within the inferential limits of the current acquisition depth, these findings provide only limited protein-level support for widespread canonical cell-death execution. The detected marker set is summarized in Table 3.

Strict differential abundance required both adjusted *p* < 0.05 and |log_2_FC| ≥ 0.585. Accordingly, ACSL4 is listed as a detected marker rather than as a strict hit because its effect size never exceeded the prespecified fold-change cutoff.

## 3. Discussion

The present study resolves the SHK-1 post-inoculation proteome into a temporally structured response characterized by a high-amplitude early perturbation followed by progressive attenuation and selective remodeling. The strongest signal emerged within the first hours after inoculation, when more than 1200 proteins met the strict differential abundance threshold at 1–2 h post-inoculation, followed by a contraction in the number of differentially abundant proteins at later time points. This pattern answers the first motivating question of the study by showing that proteome remodeling was both extensive and strongly concentrated in the early phase after bacterial exposure. Rather than reflecting a simple monotonic collapse of the host proteome, the response is better understood as a rapid early reprogramming event followed by partial adaptation, rebalancing, and residual stress across the 48 h observation window. In this context, the module-based framework adds an important layer beyond pairwise contrasts by showing that proteins with similar recurrence profiles do not follow a single trajectory but instead organize into distinct temporal programs.

This temporal structure is particularly relevant when considered in the context of previous salmonid infection studies, which have largely emphasized transcript-level responses or focused on individual innate-sensing genes rather than on coordinated protein-level remodeling [7,8,18,19]. Proteomic work in SHK-1 cells infected with a different intracellular salmonid pathogen has likewise pointed to changes in redox balance, cytoskeletal organization, vesicular systems, and metabolism, but in a distinct host–pathogen system and infection architecture [23]. The present data do not simply restate those observations at the proteomic level; instead, they define which host programs remain detectably altered at the level of protein abundance during the first 48 h after bacterial challenge. In particular, the prominence of lysosomal, vesicular, metabolic, and oxidative-stress-associated proteins is biologically consistent with macrophage-like cells confronting a facultative intracellular pathogen and supports the view that early host adaptation involves broad reorganization of cellular infrastructure rather than isolated marker responses. In the specific context of *R. salmoninarum*, the Module 1 lysosomal and vesicular signature is particularly relevant because bacterial persistence within salmonid phagocytes implies sustained host–pathogen interaction at the level of intracellular compartments, whereas the redox and antioxidant shifts captured by Modules 2 and 4 are compatible with the inflammatory and granulomatous biology associated with BKD.

The module structure suggests that several biological axes are remodeled in parallel. Module 1 was dominated by proteins associated with lysosomal proteolysis, Rab27-associated vesicular trafficking, inflammatory granule biology, and lipid-linked metabolism, including CATK, RB27B, GRN, APOC1, and AACS. This pattern is compatible with substantial remodeling of vesicular and degradative compartments after intracellular bacterial challenge [23,24]. Module 2 captured early repression followed by partial recovery of proteins linked to cytoskeletal organization, membrane-actin signaling, redox–stress integration, and xenobiotic metabolism, including ACTN1, MARCS, TXNIP, FABP7, and CYP1A, suggesting transient structural and metabolic stress rather than sustained unidirectional suppression [23,25]. Module 3 was characterized by transient induction of stress- and mitochondria-related proteins, including HSP70, CH10, CCND2, PCK2, and DLST, indicating an acute adaptive response centered on proteostasis and carbon metabolism [26,27]. Module 4, in turn, highlighted prolonged repression of antioxidant and scaffold-associated proteins, including SOD1, AKAP12, GSTT2B, TGM2, and SEPT7, followed by late rebound. Taken together, these modules indicate that the dominant signature of the dataset is broad host-cell state remodeling involving endolysosomal, metabolic, cytoskeletal, and oxidative-stress programs rather than a single pathway-centered response.

The reversals observed in Modules 1 and 4 underscore the importance of temporal sampling. Without the late 24–48 h measurements, these programs could have been misinterpreted as purely induced or purely repressed responses. Instead, the data support a model in which early perturbation is followed by selective recalibration, with some protein groups changing direction or narrowing in amplitude over time. This temporal resolution represents one of the clearest contributions of the present study, as it reveals response architecture that would remain obscured in single-endpoint analyses.

The targeted cell-death analysis should be interpreted with deliberate caution. Regulated cell-death pathways are central to host–pathogen interactions, and apoptosis, necroptosis, and pyroptosis are established components of antimicrobial responses to intracellular infection [13,14,28,29]. More recent frameworks have expanded this landscape to include ferroptosis and integrative PANoptosis, emphasizing that host-cell fate during infection is often determined by intersecting rather than isolated death programs [15,16,30,31]. However, in the present dataset the proteomic evidence was sparse and limited to reduced abundance of CASP3 and MLKL, together with sub-threshold detection of ACSL4. This result does not support a strong claim of broad execution of canonical apoptosis, necroptosis, pyroptosis, ferroptosis, or PANoptosis at the level of total protein abundance. The absence of quantified pyroptosis markers in the detected panel and the lack of concordant regulation across multiple effectors argue against overstating the existence of a dominant inflammatory cell-death program in these samples. At the same time, total-protein proteomics cannot directly resolve activation states that depend on cleavage, oligomerization, or phosphorylation. Accordingly, the observed decreases in CASP3 and MLKL should be interpreted as pathway-associated modulation rather than direct evidence of activation or inhibition of execution cascades.

The study also has important design constraints that should shape interpretation. Because only a single non-infected baseline condition was available, the analysis cannot fully disentangle infection-specific effects from time-in-culture effects. For that reason, the manuscript consistently frames the results as post-inoculation changes relative to baseline rather than as a controlled mock time-course. In addition, the infection workflow relied on PBS washing after the inoculation period and did not include an antibiotic-protection step or an independent assay to quantify residual extracellular bacteria; therefore, the post-inoculation proteome may reflect both intracellular host–pathogen interaction and residual extracellular bacterial exposure. Global label-free proteomics is also biased toward proteins that are sufficiently abundant and consistently detected, which reduces sensitivity for low-abundance regulatory nodes and post-translationally activated signaling factors. These limitations are particularly relevant for the cell-death layer, where decisive biology may occur through cleavage products or phosphorylation events that are not captured by total abundance alone. Nevertheless, the analytical strategy adopted here, including a strict differential abundance definition, explicit contrast structure, exact gene-symbol mapping, and separation between global and targeted claims, reduces the risk of false-positive biological interpretation and keeps the manuscript within the inferential limits of the experiment.

Overall, the three analytical layers converge on a coherent interpretation of the dataset. First, SHK-1 cells undergo a rapid and extensive early proteomic perturbation after *R. salmoninarum* inoculation. Second, recurrent temporal modules capture this response architecture more effectively than isolated contrasts by resolving coordinated trajectories across lysosomal, immunometabolic, cytoskeletal, stress-response, and antioxidant programs. Third, the curated cell-death layer provides only limited protein-level support for widespread canonical death-pathway execution. This interpretation is compatible with earlier evidence for intracellular persistence of *R. salmoninarum* in salmonid phagocytes and with recent evidence that salmonid innate sensing pathways engage regulated cell-death-associated signaling during intracellular bacterial challenge [6,18,19]. The main biological message of the present study is therefore not overt execution of a single canonical death pathway, but rather a structured temporal remodeling of host-cell state during the first 48 h after inoculation.

## 4. Materials and Methods

### 4.1. SHK-1 Cell Culture

The SHK-1 cell line, derived from primary cultures of adherent cells from Atlantic salmon (*Salmo salar*), was purchased from Sigma-Aldrich (Santiago, Chile; product no. 97111106-1VL), corresponding to the European Collection of Authenticated Cell Cultures SHK-1 cell line (ECACC 97111106), and used for infection time-course experiments [20,22]. Cells were challenged with the *Renibacterium salmoninarum* SF2022 strain. SHK-1 cells were maintained at 18 °C in Leibovitz’s L-15 medium (Gibco, Carlsbad, CA, USA) supplemented with 10% (*v*/*v*) fetal bovine serum (FBS, HyClone, South Logan, UT, USA) and no antibiotics. Cells were grown to 90% confluence in 25 cm^2^ flasks.

### 4.2. Cultivation and Identification of R. salmoninarum

For the in vitro assays, the *R. salmoninarum* SF2022 strain was cultured in KDM-2 medium [32] at 18 °C with shaking at 180 rpm for 5 days. Bacterial morphology and identity were confirmed by Gram staining, polymerase chain reaction (PCR), and indirect fluorescent antibody testing, following the manufacturer’s instructions.

### 4.3. In Vitro Infection Time Course

Twenty-four hours before inoculation, cultures were shifted to L-15 medium supplemented with 2% (*v*/*v*) FBS. Infection was performed in 25 cm^2^ flasks containing SHK-1 monolayers at approximately 90% confluence. Monolayers were inoculated with *R. salmoninarum* at a multiplicity of infection (MOI) of 10 CFU per cell in 3 mL of Leibovitz’s L-15 medium. After 1 h at 18 °C, cells were washed with sterile 1× PBS to reduce non-internalized bacteria. Fresh Leibovitz’s L-15 medium was then added, and samples were collected at 1, 2, 4, 6, 12, 24, and 48 h post-inoculation (hpi). A non-infected baseline group collected at T0 was included, with three biological replicates for T0 and for each post-inoculation time point. In total, 24 samples were processed: one baseline group (T0) and seven post-inoculation groups (T1, T2, T4, T6, T12, T24, and T48). Harvested cells were immediately cryopreserved in liquid nitrogen and stored at −80 °C until processing.

### 4.4. Protein Extraction

Proteins were extracted from all samples using 100 µL of lysis buffer containing 50 mM HEPES (pH 8), 1% (*w*/*v*) Triton X-100, 1% (*v*/*v*) NP-40, 1% (*v*/*v*) Tween-20, 1% (*w*/*v*) deoxycholate, 5 mM EDTA, 50 mM NaCl, 1% (*v*/*v*) glycerol, 1× complete protease inhibitor, and 5 mM DTT. Samples were incubated for 30 min at 60 °C and homogenized using an ultrasonic probe for 2 min in 10 s cycles at 40% amplitude. Proteins were alkylated with 20 mM iodoacetamide in 25 mM ammonium bicarbonate for 30 min in the dark.

Protein extracts were cleaned by chloroform/methanol precipitation [33]. One volume of extract was mixed with five volumes of methanol and one volume of chloroform, followed by three volumes of Milli-Q water (Merck Millipore, Burlington, MA, USA). Samples were centrifuged at 15,000× *g* for 5 min, the aqueous phase was removed, and the protein pellet was washed four times with 100% methanol. Pellets were dried overnight in a rotary concentrator at 40 °C.

### 4.5. Sample Preparation for Mass Spectrometry

Proteins were precipitated with five volumes of cold acetone and incubated overnight at −80 °C. Samples were then equilibrated to room temperature, centrifuged at 16,000× *g* for 15 min at 4 °C, and washed three times with cold 80% acetone. The dried pellet was resuspended in 8 M urea and 25 mM ammonium bicarbonate. Reduction was performed with DTT to a final concentration of 20 mM for 1 h at room temperature, followed by alkylation with 20 mM iodoacetamide for 1 h in the dark. Samples were diluted eightfold with 25 mM ammonium bicarbonate.

Proteolytic digestion was carried out with sequencing-grade trypsin (#V5071, Promega, Madison, WI, USA) at a 1:50 protease-to-protein ratio (*w*/*w*) for 16 h at 37 °C. Digestion was quenched with 10% formic acid. Peptides were cleaned using disposable C18 Evotip columns (EVOSEP Biosystems, Odense, Denmark) according to the manufacturer’s protocol and the general Evosep workflow [34].

### 4.6. LC-MS/MS Acquisition

Purified peptides were injected into an EVOSEP One liquid chromatography system (EVOSEP Biosystems, Odense, Denmark) coupled to a timsTOF Pro mass spectrometer (Bruker Daltonics, Bremen, Germany) using a Performance analytical column (15 cm × 150 µm, C18, 1.5 µm; EVOSEP Biosystems, Odense, Denmark). Liquid chromatography was run using the 30 samples-per-day gradient [34].

Data acquisition was controlled with TimsControl 2.0 software (Bruker Daltonics, Bremen, Germany) in PASEF mode [35] using four PASEF cycles of 0.5 s per cycle, a mass range of 100–1700 *m*/*z*, a capillary ionization voltage of 1500 V, and a capillary temperature of 180 °C. The TOF analyzer operated at 10 kHz with a resolving power of 45,000 FWHM.

### 4.7. Protein Identification and LFQ Matrix Construction

Raw data from the timsTOF Pro were processed with MSFragger v4.1 [36] within FragPipe v22.0 against the *Salmo salar* UniProt proteome [37]. Protein quantification was performed by label-free quantification using the MaxLFQ algorithm implemented in FragPipe [38]. Precursor mass tolerance was set to ±20 ppm and fragment mass tolerance to 40 ppm. Digestion parameters used trypsin specificity with a maximum of two missed cleavages. Carbamidomethylation of cysteine was defined as a fixed modification, whereas methionine oxidation and N-terminal acetylation were treated as variable modifications. Protein identifications were filtered at <1% false discovery rate using a decoy strategy, and a contaminants database was included in the search workflow.

### 4.8. Bioinformatic Analysis

#### 4.8.1. Protein Matrix Preparation

The combined protein report contained 6979 protein entries across 24 samples. MaxLFQ intensity columns were extracted from the report, zero values were converted to missing values, and protein annotations were retained from the original FragPipe export. Proteins were filtered for downstream modeling if they were detected in at least two biological replicates within at least one experimental group, yielding 6842 proteins for statistical analysis. Intensity values were log_2_-transformed and median-centered across samples [39]. The full quantified protein catalog, including detection statistics and annotation fields, is provided in Supplementary Dataset S1. All computational analyses were performed in R version 4.5.0.

#### 4.8.2. Differential Abundance Analysis

Differential abundance analysis was performed with the *limma* framework [40]. A no-intercept design matrix (~0 + group) was used to model the eight experimental groups (T0, T1, T2, T4, T6, T12, T24, and T48). Seven pairwise contrasts were specified, comparing each post-inoculation time point against the T0 baseline. Linear modeling was performed with lmFit(), followed by contrast fitting and empirical Bayes moderation with contrasts.fit() and eBayes(trend = TRUE, robust = TRUE) [41]. Multiple testing was controlled with the Benjamini–Hochberg procedure [42]. Proteins were classified as strictly differentially abundant when the adjusted *p* value was <0.05 and the absolute log_2_ fold change was at least log_2_(1.5). Volcano plots and MA plots were generated with ggplot2 [43]. The complete differential abundance output for all contrasts is reported in Supplementary Dataset S2.

#### 4.8.3. Temporal Module Analysis

To summarize recurrent post-inoculation trajectories, proteins that were significant in at least two contrasts were selected from the strict differential abundance results. Their log_2_ fold-change profiles across all seven contrasts were reshaped into a complete trajectory matrix and row-scaled. Candidate k-means solutions from k = 2 to 8 were evaluated using total within-cluster sum of squares and mean silhouette width; a four-module solution was retained as an interpretive compromise that preserved the dominant temporal programs without overfragmenting the trajectories [44]. K-means clustering was then applied with four centers, 100 random starts, and a maximum of 200 iterations. Module centroids were visualized as row-scaled temporal trajectories. A protein-level recurrence summary, the complete module membership table, and the clustering diagnostics are provided in Supplementary Datasets S3 and S4 and Figure S3, respectively. Formal over-representation analysis of temporal modules was performed with g:Profiler using *Salmo salar* as the target organism and the 6842 proteins retained for statistical modeling as the background set; GO biological process, molecular function, cellular component, KEGG, and Reactome terms were evaluated with multiple-testing correction, and results are provided in Supplementary Dataset S6.

#### 4.8.4. Curated Cell-Death Analysis

A targeted cell-death panel was manually curated to represent apoptosis, pyroptosis, necroptosis, ferroptosis, and PANoptosis. The panel comprised 46 pathway assignments corresponding to 37 unique gene symbols. Mapping to the *S. salar* proteome was performed by exact uppercase gene-symbol matching against the protein annotation table; substring matching was not used. Differential abundance statistics for matched proteins were then extracted from the global limma results. When multiple UniProt entries matched the same gene symbol, gene-level summaries retained distinct matched IDs, counted significant contrasts after deduplication and chronological ordering, reported the peak log_2_ fold change from the matched protein with the largest absolute effect size, and reported the minimum adjusted *p* value across matched proteins; Figure 3B heatmap used the mean log_2_ fold change across matched proteins within each pathway–gene–time combination. Pathway-level summaries were visualized together with a time-resolved heatmap of the detected markers. The full curated panel, including detected and undetected targets, is reported in Supplementary Dataset S5.

## 5. Conclusions

Label-free quantitative proteomics defined a reproducible baseline-referenced post-inoculation trajectory in SHK-1 cells exposed to *Renibacterium salmoninarum*. The study shows that the response is dominated by three linked features: a high-amplitude early perturbation of the proteome, four recurrent temporal modules that resolve the dominant architecture of remodeling, and only limited protein-level support for widespread canonical cell-death-pathway execution. The dataset therefore supports a conservative model in which early host-cell remodeling, rather than overt death-program execution, is the predominant proteomic signature of the SHK-1 response during the first 48 h after inoculation. Future work should pair time-matched mock controls with targeted validation of caspase processing, MLKL activation, and ferroptosis-associated lipid peroxidation to resolve which components of this remodeling are infection-specific and which converge on executable cell-death pathways.

## Figures and Tables

**Table 1:** Summary of strict differentially abundant proteins across the post-inoculation time course.

Comparison	Total Proteins Tested	Strict DE Proteins	Up-Regulated	Down-Regulated
SHK_T1 vs. SHK_T0	6842	1281	412	869
SHK_T2 vs. SHK_T0	6842	1296	407	889
SHK_T4 vs. SHK_T0	6842	902	374	528
SHK_T6 vs. SHK_T0	6842	704	339	365
SHK_T12 vs. SHK_T0	6842	775	398	377
SHK_T24 vs. SHK_T0	6842	432	242	190
SHK_T48 vs. SHK_T0	6842	391	194	197

**Table 2:** Selected recurrent proteins representing the four temporal modules. Module 1, sustained induction followed by late reversal; Module 2, early repression followed by recovery/induction; Module 3, transient induction followed by late negative phase; Module 4, sustained repression with late rebound. Protein labels are reported as annotated in the search database.

Module	Protein Label	Protein Description	Functional Context	Peptides	Strict Contrasts (*n*)	Peak log_2_FC
Module 1	CATK	Cathepsin K	Lysosomal proteolysis	13	7	3.8413
Module 1	APOC1	Apolipoprotein C-I	Lipid-associated immunometabolism	16	5	2.0813
Module 1	AACS	Acetoacetyl-CoA synthetase	Acetyl-CoA and lipid metabolism	28	5	1.6451
Module 1	RB27B	Ras-related protein Rab-27B	Vesicular trafficking and granule dynamics	7	7	1.9512
Module 1	GRN	Granulin	Granule-associated inflammatory signaling	6	7	3.1223
Module 2	CYP1A	Cytochrome P450 1A	Xenobiotic and oxidative metabolism	14	6	3.2932
Module 2	ACTN1	Alpha-actinin-1	Actin cytoskeleton organization	92	5	−1.3536
Module 2	MARCS	MARCKS-like protein	PKC-associated membrane-actin signaling	9	6	−2.3897
Module 2	FABP7	Fatty acid-binding protein 7	Fatty-acid handling	11	3	−1.0388
Module 2	TXNIP	Thioredoxin-interacting protein	Redox–stress integration	12	6	−1.0342
Module 3	HSP70	Heat shock cognate 70 kDa protein	Proteotoxic stress response	64	6	1.9404
Module 3	CH10	10 kDa mitochondrial chaperonin	Mitochondrial chaperone response	11	2	0.6429
Module 3	CCND2	G1/S-specific cyclin D2	Cell-cycle regulation	5	3	1.2701
Module 3	PCK2	Phosphoenolpyruvate carboxykinase (GTP)	Anaplerotic carbon metabolism	16	5	1.0421
Module 3	DLST	2-oxoglutarate dehydrogenase E2 component	Mitochondrial carbon metabolism	6	6	1.3952
Module 4	SOD1	Cu/Zn superoxide dismutase	Antioxidant defense	6	7	−2.7288
Module 4	SEPT7	Septin 7	Cytoskeletal scaffold remodeling	25	5	−1.0404
Module 4	AKAP12	A-kinase anchor protein 12	Signal-scaffold organization	17	6	−3.3023
Module 4	GSTT2B	Glutathione S-transferase theta	Glutathione-dependent detoxification	10	4	−1.0871
Module 4	TGM2	Transglutaminase 2	Stress-associated protein crosslinking	45	5	−1.0425

**Table 3:** Detected cell-death markers recovered from the targeted curated panel.

Gene Symbol	Assigned Pathways	Strict DE	Strict Contrasts	Peak log_2_FC	Minimum Adjusted *p* Value
CASP3	Apoptosis	Yes	T1; T2; T4; T48	−1.0341	1.932 × 10^−7^
MLKL	Necroptosis; PANoptosis	Yes	T1; T12	−0.6854	1.356 × 10^−4^
ACSL4	Ferroptosis	No		0.2736	8.332 × 10^−4^

## Data Availability

The data presented in this study are available in the article and Supplementary Materials. Further inquiries can be directed to the corresponding authors.

## Supplementary Materials

The following supporting information can be downloaded at: https://www.mdpi.com/article/10.3390/ijms27135773/s1.

## Abbreviations

The following abbreviations are used in this manuscript:

BKD	Bacterial kidney disease
CFU	Colony-forming units
DTT	Dithiothreitol
EDTA	Ethylenediaminetetraacetic acid
FBS	Fetal bovine serum
FC	Fold change
FDR	False discovery rate
FWHM	Full width at half maximum
HEPES	4-(2-Hydroxyethyl)-1-piperazineethanesulfonic acid
hpi	Hours post-inoculation
LC-MS/MS	Liquid chromatography–tandem mass spectrometry
LFQ	Label-free quantification
MOI	Multiplicity of infection
PASEF	Parallel accumulation–serial fragmentation
PBS	Phosphate-buffered saline
PCA	Principal component analysis
PCR	Polymerase chain reaction
