# Supplementary material for: Time-Resolved Label-Free Proteomics of SHK-1 Cells After Renibacterium salmoninarum Inoculation Reveals Early Host-Cell Remodeling
Source: Int J Mol Sci. 2026 Jun 26;27(13):5773. doi: 10.3390/ijms27135773 (PMC13360928; doi:10.3390/ijms27135773)
Supplement: Supplementary file 1 [file ijms-27-05773-s001.zip › Supplementary_Material_Legends.pdf]

Supplementary figures

Supplementary Figure S1. Sample-level principal component analysis of the normalized proteomic matrix. PCA was computed using 5,034 proteins quantified across all 24 samples after log2 transformation and median-centering. Panel A shows PC1 versus PC2, with samples labeled by replicate, colored by time point, and shaped by experimental status. PC1 and PC2 explained 32.2% and 16.7% of total variance, respectively. Panel B shows the variance explained by the first eight principal components, with bars indicating per-component variance and the red line indicating cumulative explained variance.

Supplementary Figure S2. Heatmap of all proteins meeting the strict differential-abundance threshold in at least one post-inoculation contrast. The heatmap includes 2,254 unique proteins tested across the seven baseline-referenced contrasts against T0. Columns represent SHK_T1, SHK_T2, SHK_T4, SHK_T6, SHK_T12, SHK_T24, and SHK_T48 versus SHK_T0, and cell colors indicate log2 fold changes relative to T0, with red and blue denoting increased and decreased abundance, respectively. Rows are grouped by temporal module when proteins were significant in at least two contrasts and assigned to the four-module k-means solution; proteins significant in only one contrast are shown as a separate single-contrast group.

Supplementary Figure S3. Clustering diagnostics used to select the temporal-module solution. Candidate k-means solutions from $k = 2$ to $k = 8$ were evaluated using total within-cluster sum of squares and mean silhouette width. Panel A shows the elbow profile, where the four-cluster solution is highlighted. Panel B shows the corresponding mean silhouette profile. The four-module solution was retained as an interpretive compromise that preserved the major temporal response programs without overfragmenting the protein trajectories.

Supplementary datasets

Supplementary Dataset S1. Quantified protein catalog used for downstream proteomic analysis. The dataset contains 6,979 protein entries from the combined FragPipe/MaxLFQ protein report, including protein identifiers, gene names, descriptions, protein length, organism annotation, peptide counts, protein probability, sample-level and group-level detection breadth, missing-value percentage, summary $\log_2$ intensity metrics, and the detectability-filter flag. A total of 6,842 proteins passed the detectability filter and were retained for statistical modeling.

Supplementary Dataset S2. Complete differential-abundance output for all seven post-inoculation contrasts relative to T0. The dataset contains 47,894 protein-contrast rows, corresponding to 6,842 tested proteins across seven pairwise comparisons. For each protein and contrast, the table reports $\log_2$ fold change, average expression, moderated t statistic, raw and adjusted p values, B statistic, annotation fields, comparison label, regulation class, and strict-significance status. Strict differential abundance was defined as adjusted $p < 0.05$ and $|\log_2 FC| \geq 0.585$. Across all contrasts, 2,254 unique proteins met the strict threshold at least once.

Supplementary Dataset S3. Protein-level recurrence summary for all unique strict differentially abundant proteins. The dataset contains 2,254 proteins that met the strict differential-abundance threshold in at least one contrast. For each protein, the table reports the number of significant contrasts, significant time points, up- and down-regulated contrasts, first and last significant post-inoculation time points, peak and maximum

absolute $\log_2$ fold change, minimum adjusted p value, mean $\log_2$ fold change, dominant directionality, and temporal-module assignment when applicable. Proteins significant in at least two contrasts were eligible for temporal-module analysis, whereas single-contrast proteins are retained without module assignment.

Supplementary Dataset S4. Temporal module membership table for recurrent differentially abundant proteins. The dataset contains 1,278 proteins significant in at least two post-inoculation contrasts and assigned to one of four k-means temporal modules. For each protein, the table reports module identity, number and identity of significant contrasts, peak $\log_2$ fold change, maximum absolute $\log_2$ fold change, minimum adjusted p value, correlation with the assigned module centroid, and module trajectory annotation. Module sizes were 311 proteins in Module 1, 498 in Module 2, 137 in Module 3, and 332 in Module 4.

Supplementary Dataset S5. Curated cell-death marker panel used for targeted pathway-level interrogation of the global proteomic dataset. The panel contains 46 pathway assignments corresponding to 37 unique gene symbols across apoptosis, pyroptosis, necroptosis, ferroptosis, and PANoptosis. For each pathway-gene assignment, the table reports priority, functional theme, annotation notes, matched UniProt identifiers, matched gene names, detection status in the proteome, strict differential-abundance status, number and identity of significant contrasts, peak $\log_2$ fold change, and minimum adjusted p value. Only CASP3, MLKL, and ACSL4 were detected in the proteomic dataset; CASP3 and MLKL met the strict differential-abundance threshold, whereas ACSL4 remained below the predefined fold-change cutoff.

Supplementary Dataset S6. Functional enrichment results for the four recurrent temporal modules. Over-representation analysis was performed with g:Profiler using Salmo salar as the target organism and the 6,842 proteins retained for statistical modeling as the background set. The dataset reports significant GO, KEGG, and Reactome terms by module, including source, term identifier, term name, p value, term size, query size, intersection size, precision, recall, and parent term information. Formal enrichment supported the lysosomal interpretation of Module 1, including KEGG lysosome biogenesis, whereas Modules 2-4 did not yield significant GO, KEGG, or Reactome terms after multiple-testing correction.
