# Supplementary figures and images for: Time-Resolved Label-Free Proteomics of SHK-1 Cells After Renibacterium salmoninarum Inoculation Reveals Early Host-Cell Remodeling
Source: Int J Mol Sci. 2026 Jun 26;27(13):5773. doi: 10.3390/ijms27135773 (PMC13360928; doi:10.3390/ijms27135773)

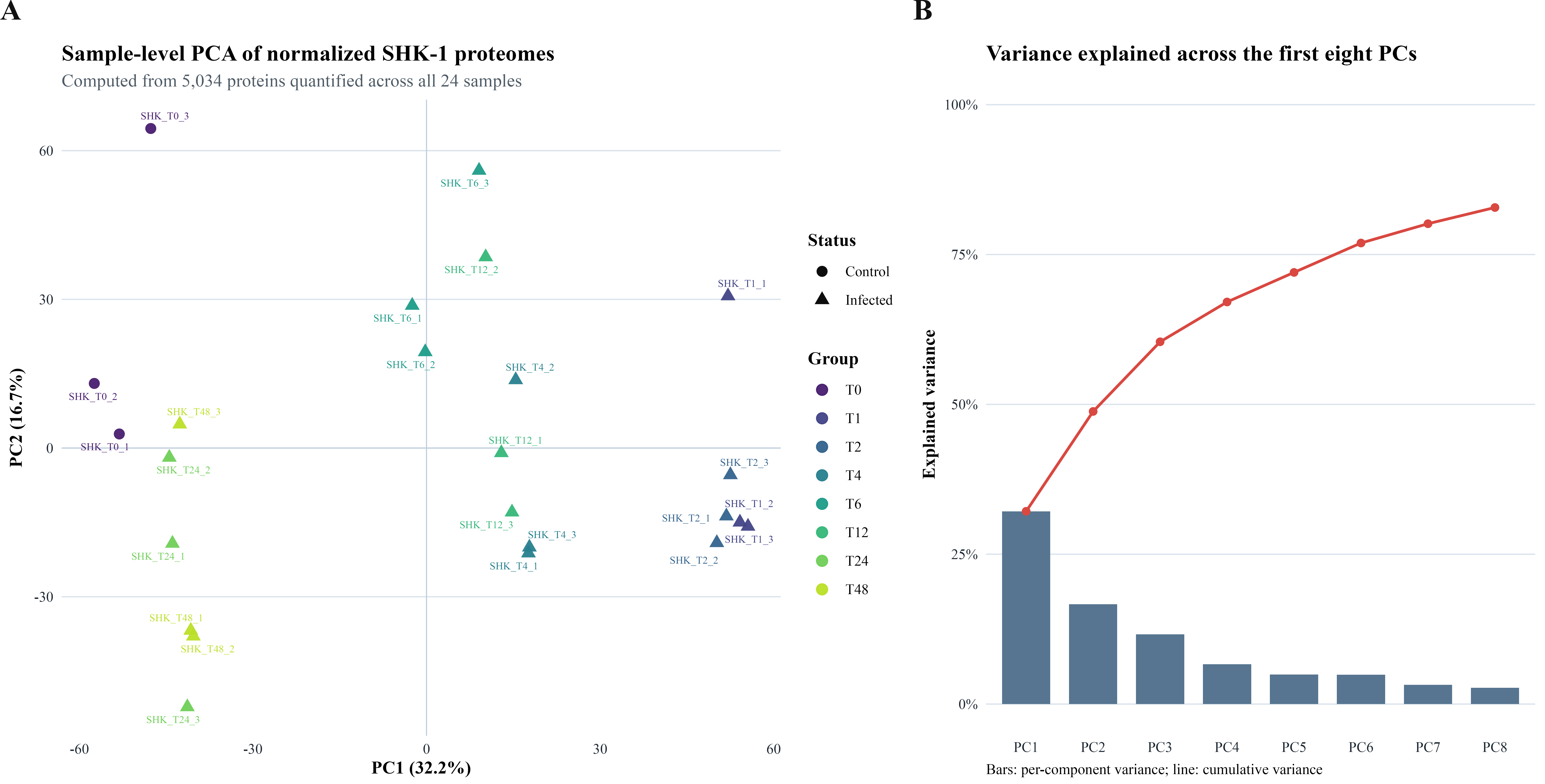

Supplement: Supplementary file 1 [file ijms-27-05773-s001.zip › Figure_Supplementary_Figure S1.png]

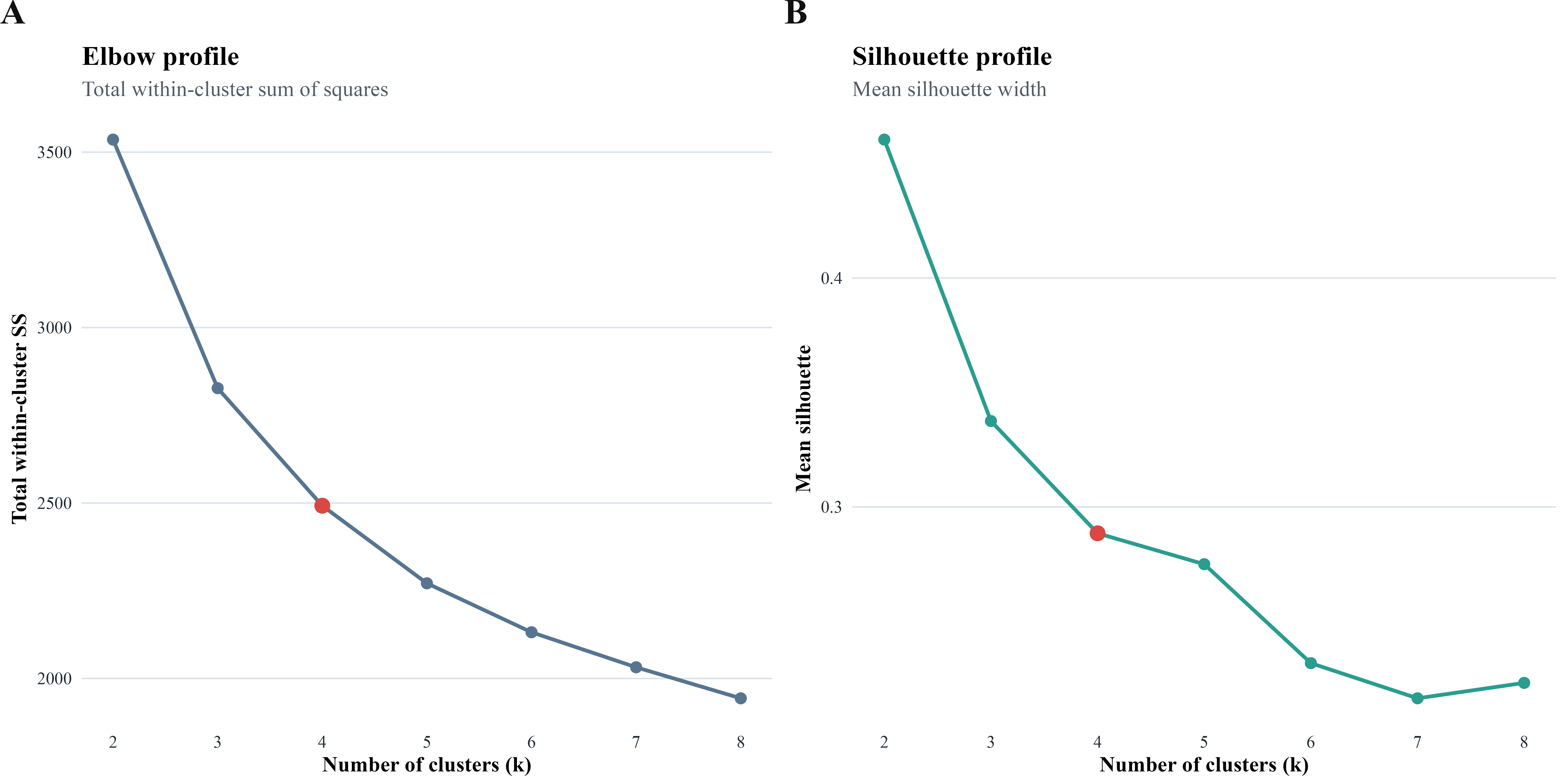

Supplement: Supplementary file 1 [file ijms-27-05773-s001.zip › Figure_Supplementary_Figure S3.png]
